# Isolated iliac artery aneurysm in association with congenital pelvic kidney treated with iliac branch device: case report

**DOI:** 10.1186/s13019-021-01409-x

**Published:** 2021-03-17

**Authors:** Guilherme Centofanti, Kenji Nishinari, Bruna De Fina, Rafael Noronha Cavalcante, Mariana Krutman, Ross Milner

**Affiliations:** 1 Hospital Alemão Osvaldo Cruz, R. Treze, de Maio, 1815, São Paulo, 01323-020 Brazil; 2 Cava endovascular, Paulista Ave. 91, Suite 909, São Paulo, SP 01311-000 Brazil; 3 The University of Chicago Medicine & Biological Sciences, 5841 S. Maryland Ave., Rm. J-555, MC5028, Chicago, IL 60637 USA

**Keywords:** Pelvic kidney, Isolated iliac artery aneurysm, Iliac branch device, Case report

## Abstract

**Background:**

Association of abdominal aortic aneurysm with congenital pelvic kidney is rare and association with isolated iliac artery aneurysm is not yet described in the literature.

**Case presentation:**

We present a case of successful repair of an isolated common iliac artery aneurysm associated with a congenital pelvic kidney treated by an endovascular technique. A 75-year-old man was referred for the treatment of an asymptomatic left common iliac artery aneurysm. A computed tomography angiography revealed an isolated left common iliac artery aneurysm and a left pelvic kidney. The maximum diameter of the aneurysm was 32 mm. The congenital pelvic kidney was supplied by three small superior polar arteries that emerged from the proximal non-aneurysmal portion of the common iliac artery and the main artery that arose from the left internal iliac artery. The aneurysm exclusion was accomplished by using an iliac branch device (Gore Excluder Iliac Branch, Flagstaff, AZ). The 1 and 6 months computed tomography angiography after the procedure demonstrated complete exclusion of the aneurysm and preservation of all renal arteries.

**Conclusion:**

Treating patients with an association of iliac artery aneurysms and pelvic kidneys can be a challenge due the variable arterial anatomy. The use of iliac branch device is a safe and effective alternative in selected cases.

## Background

Renal vascular anomalies can be associated with an ectopic or horseshoe kidney [1]. The incidence of pelvic kidneys is between 1 out of 2100 and 3000 births in the population [2] and it results from failure of the embryological kidney to ascend during the fourth to eighth week of gestation [3].

Association of abdominal aortic aneurysm with congenital pelvic kidney is rare and association with isolated iliac artery aneurysm is not yet described in the literature. There are few reports about endovascular aortic aneurysm repair and congenital pelvic kidney [4].

We present a case of successful repair of an isolated common iliac artery (CIA) aneurysm associated with a congenital pelvic kidney treated by an endovascular technique.

## Case presentation

**Fig. 1:**
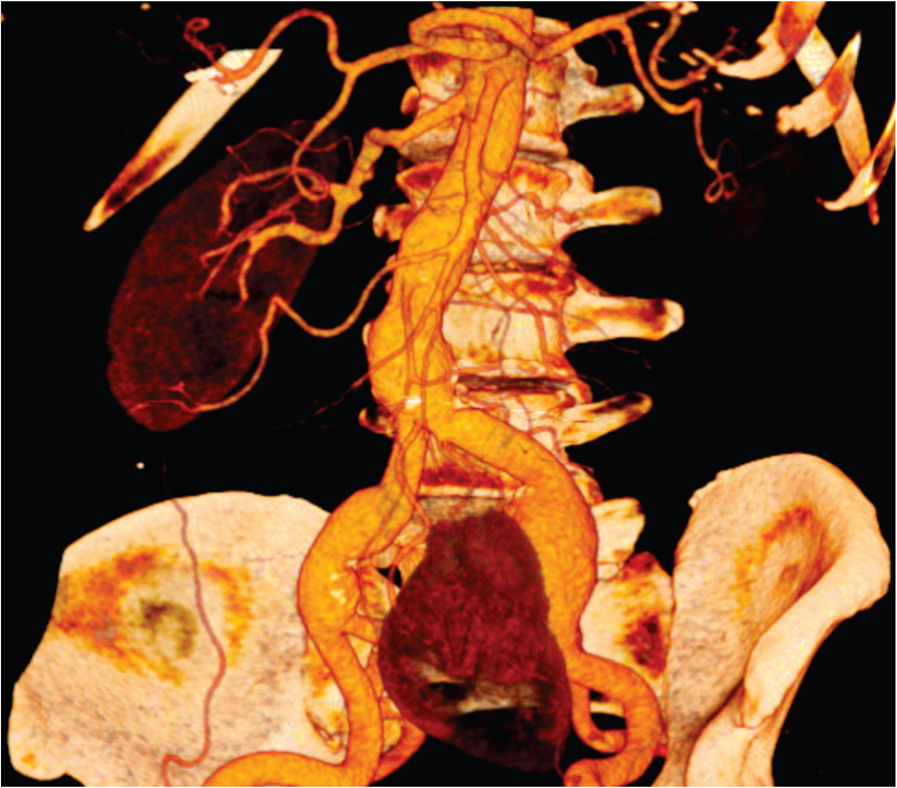
Reconstructed three-dimensional computed tomography image showing the position of the kidneys

**Fig. 2:**
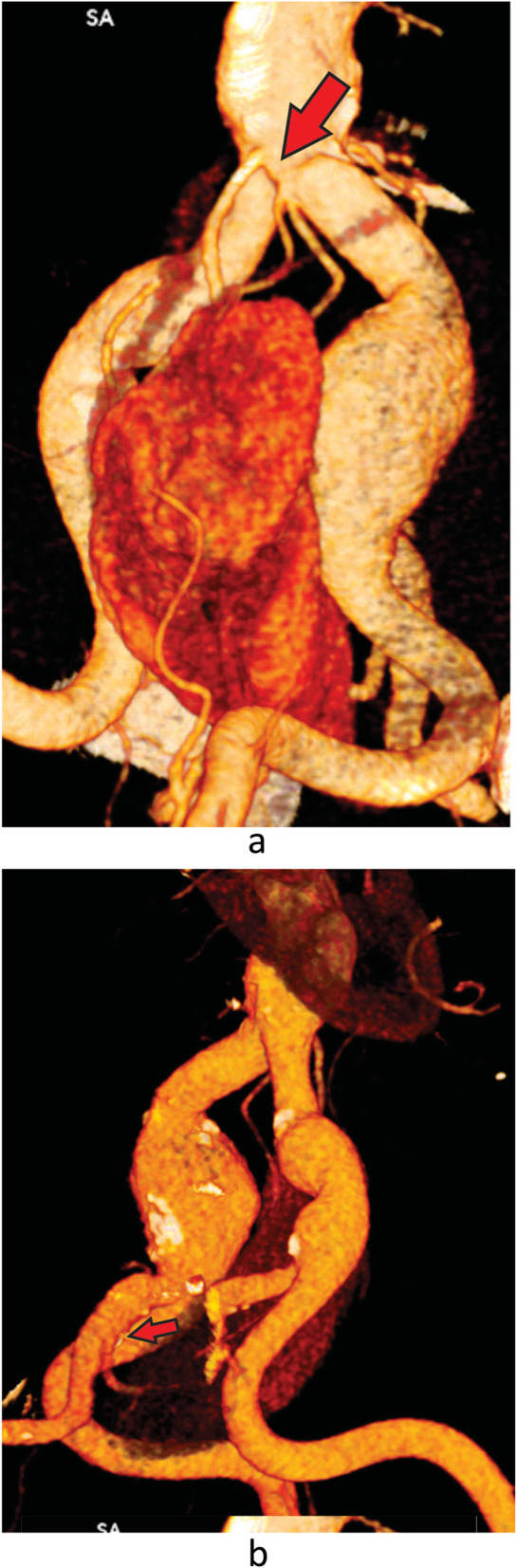
**a** Reconstructed three-dimensional computed tomography image showing preoperative aneurysm and the pelvic kidney. The polar arteries are visualized. Red arrow. **b:** Reconstructed three-dimensional computed tomography image showing preoperative aneurysm and the pelvic kidney. The predominant artery that arose from the internal iliac artery are visualized. Red arrow

**Fig. 3:**
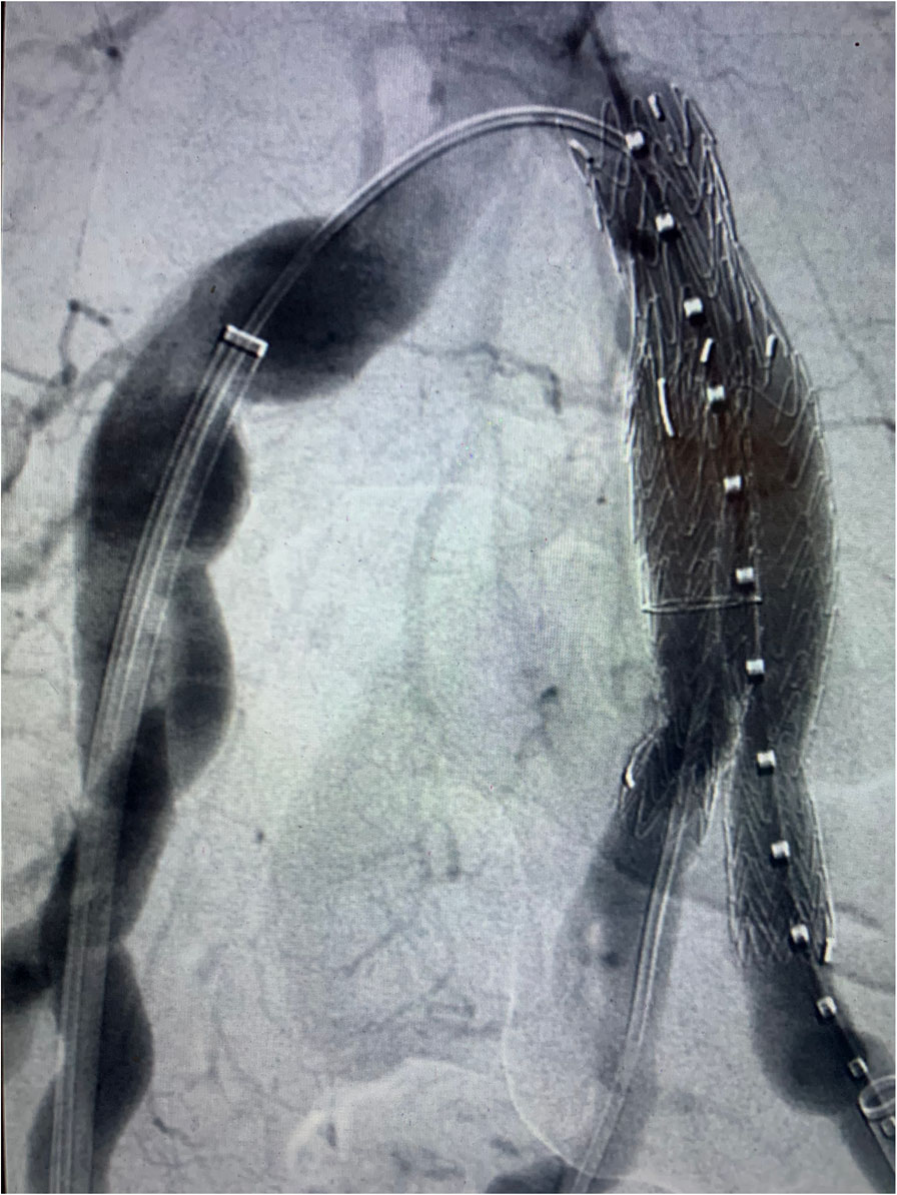
Intra operative image showing the aneurysm exclusion with iliac branch device (Gore Excluder Iliac Branch, Flagstaff, AZ) without aortic stent graft

A 75-year-old man was admitted for the treatment of an asymptomatic left CIA aneurysm. The aneurysm was diagnosed during a routine abdominal ultrasound. A computed tomography angiography (CTA) revealed an isolated left CIA aneurysm and a left pelvic kidney (Fig. 1). The maximum diameter of the CIA aneurysm was 32 mm. The congenital pelvic kidney was supplied by three small superior polar arteries that emerged from the proximal non-aneurysmal portion of the CIA and the main artery that arose from the internal iliac artery (Fig. 2a and b). The distance between the superior polar arteries and the beginning of the aneurysm was 28,9 mm. The special issues about this case was to preserve all of the blood supply of the pelvic kidney and exclude the aneurysm. It was accomplished by using an iliac branch device (IBD) (Gore Excluder Iliac Branch, Flagstaff, AZ) without an aortic stent graft (Fig. 3). Preoperative serum blood urea nitrogen and creatinine levels were 22 and 1,03 mg/dl, respectively. There was no change in the postoperative period.

After induction of general anesthesia, we performed the Preclose technique via bilateral femoral artery access, guided by ultrasound, with two Perclose Proglide (Abbott Vascular) for each site. After achieving access, the patient was systematically heparinized. A 16Fr Dry Seal Flex Introducer Sheath (Gore, Flagstaff, AZ) was advanced over an Extra Stiff Wire Guide in the ipsilateral site, and a 12Fr Dry Seal Flex Introducer Sheath (Gore, Flagstaff, AZ) was advanced over an Extra Stiff Wire Guide in the contralateral site. A vertebral catheter and a hydrophilic guidewire were introduced via the 16Fr Sheath, and an EN Snare (Merit Medical System, Malvern, PA) was introduced in the contralateral 12Fr Sheath, establishing the through-and-through femoral access. After, we advanced the IBD (23x12x10 mm) into position over both wires. The first step of deployment was done, releasing the iliac branch portal. The 12 Fr Sheath was advanced up and over the aortic bifurcation using a pushing and pull movement and the catheterization of the internal iliac artery was performed with a vertebral catheter and hydrophilic guidewire, with was later replaced by an Amplatz. Support Wire Guide 1 cm short tip that allowed to advance the internal iliac component (16x14x10 mm) into the internal iliac artery. The iliac branch portal was dilated using a 14 mm angioplasty balloon Zeppelin (Scitech, Aparecida de Goias, GO). The second step of the IBD was done with the release of the external iliac branch. After, kissing-balloon angioplasty was performed using a Giant Balloon Catheter (Scitech, Aparecida de Goias, GO) for the external iliac component. Completion angiography was performed demonstrating exclusion of the aneurysm, preservation off all renal arteries without endoleaks.

**Fig. 4:**
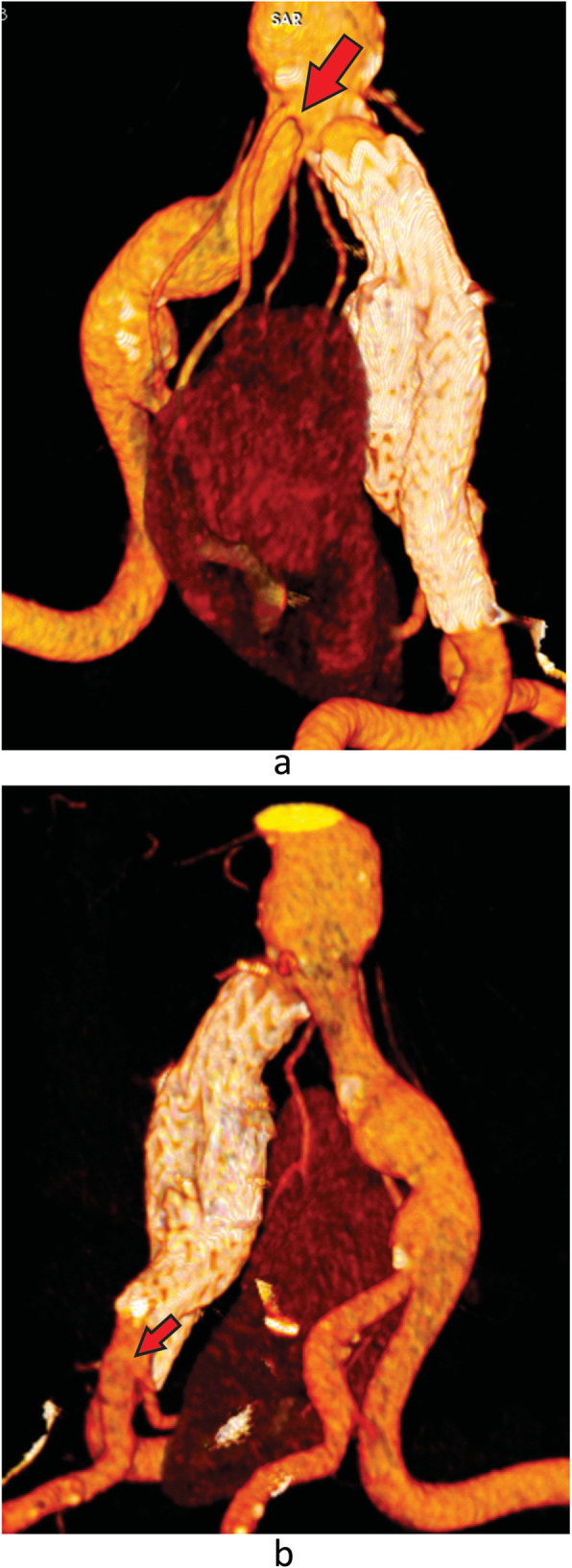
**a** 6 months postoperative three-dimensional reconstruction computed tomography showing proper positioning of the iliac branch device and preservation of the polar arteries. Red arrow. **b** 6 months postoperative three-dimensional reconstruction computed tomography showing proper positioning of the iliac branch device and preservation of the predominant renal artery. Red arrow

A CTA was performed 1 and 6 months after the procedure and demonstrated complete exclusion of the aneurysm and preservation of all renal arteries (Fig. 4a and b).

## Discussion

Various congenital renal anomalies have been reported and a congenital pelvic kidney is the most uncommon of the six types of renal ectopia (pelvic, lumbar, abdominal, cephalad, thoracic and crossed) [5]. The vascular supply for the kidney is anomalous and the arteries usually arise from the aortic bifurcation, common iliac artery, external iliac artery and rarely from inferior mesenteric artery [3]. In our case, the main artery emerged from the internal iliac artery, which makes the case extremely rare and the preservation of the internal iliac artery essential.

The combination of congenital pelvic kidney and aortic aneurysm was reported in a radiological study which demonstrated that 0,18% of patients who underwent a major aortic surgery had pelvic kidneys [6]. The technical challenge, besides excluding the aneurysm, is to preserve renal arterial supply and renal function. Coverage of accessories renal arteries is usually well tolerated and does not lead to significant impairment of renal function [7]. But, postoperative renal infarction was detected in the vast majority of patients [8]. The treatment strategy is based on the anatomy of each patient and attempting to preserve the totality of renal arteries when possible.

The aim of surgical treatment of iliac aneurysm is to avoid rupture and preserve renal function in this particular case. Before the advent of endovascular repair, open surgery was the mainstay of treatment [9]. But with a significant decrease in morbidity and mortality, and with a fewer complications, in patients with iliac artery aneurysm endovascular repair may be considered as first line therapy [9]. In our case, we had favorable anatomy to preserve all of the blood supply to the pelvic kidney using an IBD. IBDs represent the main option to preserve antegrade flow to internal iliac artery when anatomically feasible, with estimated rates of freedom from target IBD occlusion and freedom from target hypogastric artery occlusion, in 60 months, 86% and 98,3% respectively [10].

Instruction for use of IBDs recommend the use of a proximal bifurcated aortic device. However, when an adequate sealing zone is found in the CIA, an isolated iliac branch implant could be a treatment option without affecting perioperative and long-term results [10]. The most important anatomical requirement is the presence of a suitable sealing zone length in the proximal CIA of 10 mm or greater [10].

## Conclusion

Treating patients with an association of iliac artery aneurysms and pelvic kidneys can be a challenge due the variable arterial anatomy. The use of IBDs is a safe and effective alternative in selected cases.

## Data Availability

Not applicable.

## Abbreviations

CIA: Common Iliac Artery
CTA: Computed Tomography Angiography
IBD: Iliac Branch Device

## References

[1] Bauer SB, Perlmutter AD, Retik AB, Walsh PC, Retik AB, Stamey TA, Vaughan ED (1992). Anomalies of the upper urinary tract. Campbell’s urology.

[2] Merklin RJ, Michels NA (1958). The variant renal and suprarenal blood supply with data on the inferior phrenic, ureteral and gonadal arteries: a statistical analysis based on 185 dissections and review of the literature. J Int Coll Surg.

[3] Marone EM, Tshomba Y, Brioschi C, Calliari FM, Chiesa R (2008). Aortoiliac aneurysm associated with congenital pelvic kidney: a short series of successful open repairs under hypothermic selective renal perfusion. J Vasc Surg.

[4] Date K, Okada S, Ezure M, Takihara H, Okonogi S, Hasegawa Y, Sato Y, Kaneko T (2015). Aortoiliac aneurysm with congenital right pelvic kidney. Heart Vessel.

[5] Morales JP, Greemberg RK (2009). Customised stent graft for complex Thoraco-abdominal aneurysm associated with congenital pelvic kidney. Eur J Vasc Endovasc Surg.

[6] Faggioli G, Freyrie A, Pilato A, Ferri M, Curti T, Paragona O, D’Addato M (2003). Renal anomalies in aortic surgery: contemporary results. Surgery.

[7] Karmacharya J, Parmer SS, Antezana JN, Fairman RM, Woo EY, Velazquez OC, Golden MA, Carpenter JP (2006). Outcomes of accessory renal artery occlusion during endovascular aneurysm repair. J Vasc Surg.

[8] Greenberg JI, Dorsey C, Dalman RL, Lee JT, Harris EJ, Hernandez-Boussard T, Mell MW (2012). Long-term results after accessory renal artery coverage during endovascular aortic aneurysm repair. J Vasc Surg.

[9] Wanhainen A, Verzini F, Herzeele IV, van Herzeele I, Allaire E, Bown M, Cohnert T, Dick F, van Herwaarden J, Karkos C, Koelemay M, Kölbel T, Loftus I, Mani K, Melissano G, Powell J, Szeberin Z, ESVS Guidelines Committee, de Borst GJ, Chakfe N, Debus S, Hinchliffe R, Kakkos S, Koncar I, Kolh P, Lindholt JS, de Vega M, Vermassen F, Document reviewers, Björck M, Cheng S, Dalman R, Davidovic L, Donas K, Earnshaw J, Eckstein HH, Golledge J, Haulon S, Mastracci T, Naylor R, Ricco JB, Verhagen H (2019). ESVS 2019 management guidelines for abdominal Aorto-iliac artery aneurysms. Eur J Vasc Endovasc Surg.

[10] Fargion AT, Masciello F, Pratesi C, Pratesi G, Torsello G, Donas KP (2018). Results of the multicenter pELVIS registry for isolated common iliac artery aneurysms treated by the iliac branch device. J Vasc Surg.

